# Neuropathogenesis of a highly pathogenic avian influenza virus (H7N1) in experimentally infected chickens

**DOI:** 10.1186/1297-9716-42-106

**Published:** 2011-10-07

**Authors:** Aida J Chaves, Núria Busquets, Rosa Valle, Raquel Rivas, Júlia Vergara-Alert, Roser Dolz, Antonio Ramis, Ayub Darji, Natàlia Majó

**Affiliations:** 1 Centre de Recerca en Sanitat Animal (CReSA), UAB-IRTA, Campus de la Universitat Autónoma de Barcelona, 08193 Bellaterra, Barcelona, Spain; 2 Departament de Sanitat i Anatomia Animals, Universitat Autónoma de Barcelona, 08193 Bellaterra, Barcelona, Spain

## Abstract

In order to understand the mechanism of neuroinvasion of a highly pathogenic avian influenza virus (HPAIV) into the central nervous system (CNS) of chickens, specific pathogen free chickens were inoculated with a H7N1 HPAIV. Blood, cerebrospinal fluid (CSF), nasal cavity and brain tissue samples were obtained from 1 to 4 days post-inoculation (dpi) of infected and control chickens. Viral antigen topographical distribution, presence of influenza A virus receptors in the brain, as well as, the role of the olfactory route in virus CNS invasion were studied using different immunohistochemistry techniques. Besides, viral RNA load in CSF and blood was quantified by means of a quantitative real-time reverse transcription-polymerase chain reaction. Viral antigen was observed widely distributed in the CNS, showing bilateral and symmetrical distribution in the nuclei of the diencephalon, mesencephalon and rhombencephalon. Viral RNA was detected in blood and CSF at one dpi, indicating that the virus crosses the blood-CSF-barrier early during infection. This early dissemination is possibly favoured by the presence of Siaα2,3 Gal and Siaα2,6 Gal receptors in brain vascular endothelial cells, and Siaα2,3 Gal receptors in ependymal and choroid plexus cells. No viral antigen was observed in olfactory sensory neurons, while the olfactory bulb showed only weak staining, suggesting that the virus did not use this pathway to enter into the brain. The sequence of virus appearance and the topographical distribution of this H7N1 HPAIV indicate that the viral entry occurs via the haematogenous route, with early and generalized spreading through the CSF.

## Introduction

Influenza A viruses (IAV) are important pathogens that infect a wide range of avian and mammal species around the world [1]. Moreover, they are able to infect humans causing upper respiratory disease and sporadically more severe health complications, such as pneumonia and central nervous system (CNS) dysfunction [2]. In birds, IAV produce a variety of disease symptoms and, according to the severity, they are classified as low pathogenic avian influenza viruses (LPAIV) or highly pathogenic avian influenza viruses (HPAIV) [3]. LPAIV include those viruses that induce only a slight or asymptomatic infection, whereas, HPAIV cause a generalized infection where oedema, haemorrhages, and multiple organ failure are common findings [4]. This classification is mainly determined by the presence of multiple basic amino acids in the haemagglutinin cleavage site in the HPAIV, which mainly include viruses belonging to the H5 and H7 subtypes [3].

A large amount of the reported natural and experimental HPAIV infections in birds describes CNS as one of the main target organs affected during the disease [4-6]. Different pathways for IAV to reach the CNS have been hypothesized such as through the peripheral nervous system [7,8], via the olfactory nerves [9], or through the bloodstream [10]. In the mouse model, the virus reaches the brain through trans-synaptic invasion via cranial nerves [9,10]. In chickens, the lesion profile reported in the literature points up to viraemia and alterations of the vascular endothelium as the mechanism of virus dissemination and damage to the CNS [5,6,11,12]. In fact, previous studies in natural and experimental HPAIV infections have demonstrated the association between the severity of the lesions and the affinity of the virus for endothelial cells in specific tissues, indicating that the endothelial tropism has a central role in the pathogenesis [4,5,13,14].

The aim of this study was to elucidate the entry point of a H7N1 HPAIV into the CNS of chickens and to define factors determining cell tropism within the brain. For that purpose, the chronological and topographical distribution of viral antigen, as well as the presence and distribution of IAV receptors in the CNS of infected chickens was established. A double immunostaining was employed to determine the role of the olfactory sensory neurons (OSN) in the neuropathogenesis as an initial target of IAV entry into the CNS. Lastly, the presence of haematogenous dissemination was determined by means of viral RNA detection in the blood and cerebrospinal fluid (CSF) using a quantitative real-time reverse transcription-polymerase chain reaction (RT-qPCR).

## Materials and methods

### Virus

The avian influenza virus used in this study consisted of a sixth passage A/chicken/Italy/5093/99 H7N1, kindly provided by Dr Ana Moreno from the Istituto Zooprofilattico Sperimentale della Lombardia e dell' Emilia Romagna in Brescia, Italy. The intravenous pathogenicity index (IVPI) of this virus was 2.8, indicating that it is a highly pathogenic strain. This virus was propagated in 9 to 11-day-old specific pathogen free (SPF) embryonated chicken eggs. The fifty percent embryo lethal dose (ELD_50_) was carried out in SPF embryonated eggs and was determined as described previously [15].

### Chickens and experimental infection

Twenty-nine SPF chickens (Charles River, SPAFAS, MA, USA), were hatched and subsequently placed in negative pressure isolators under biosafety level 3 (BSL-3) containment conditions at the Centre de Recerca en Sanitat Animal (CReSA). At 15 days of age, chickens were randomly divided into two groups. The first group consisted of 17 chickens that were inoculated intranasally with 50 μL diluted infectious allantoic fluid containing 10^6 ^ELD_50 _H7N1 HPAIV. The second group consisted of 12 chickens that were inoculated with PBS and used as negative controls. Chickens were monitored daily by visual observation for clinical signs. At 1, 2, 3, and 4 dpi, 3 chickens from each group were randomly selected for necropsy and sampled. In addition, chickens found dead on these same days were necropsied and included in the study, obtaining 4 and 1 additional chickens at 3 and 4 dpi, respectively. Blood samples were only taken from 3 chickens from each group at 1 and 3 days post inoculation (dpi) in 1 mL Alsever's solution (Sigma-Aldrich, Madrid, Spain) to determine the presence of viral RNA in the bloodstream by RT-qPCR. All chickens were kept and managed according to procedures reviewed and approved by the Ethics Committee for Animal and Human Experimentation of the Universitat Autònoma de Barcelona.

### Post-mortem sampling

CSF samples were collected from 3 euthanized chickens from each group at 1 and 3 dpi. Briefly, the skin and muscle over the atlanto-occipital joint were carefully removed. Later, the skull and the duramater over the brain and spinal cord were separated in anterior direction. After that, roughly 1 μL of CSF was collected from the *cisterna magna *using a micropipette. These samples were used to detect the presence of viral RNA by RT-qPCR.

**Figure 1:**
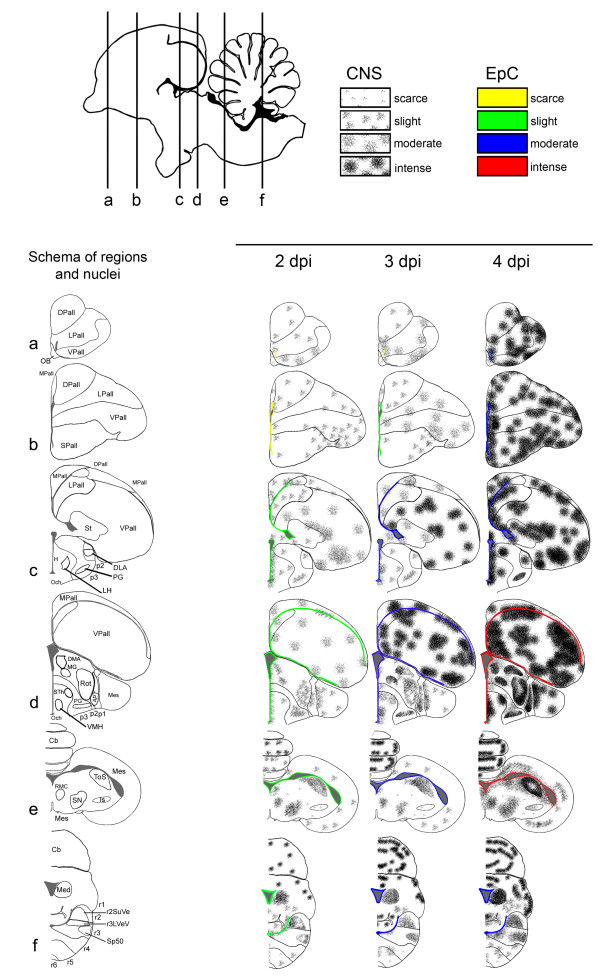
**Schematic drawing to show the topographical distribution of IAV antigen in chickens infected with H7N1 HPAIV at 2, 3 and 4 dpi**. A. Schematic view of a chicken's brain showing the rostro-caudal levels depicted in the coronal diagrams below. The diagram on the left shows the brain regions evaluated in each section and on sections c, d, e, and f, the neural nuclei where viral antigen was found bilaterally and symmetrically are represented and labelled. The severity of viral antigen immunostaining in cells of the central nervous system (neurons, endothelial cells and microglia) is represented using different density of dots, as follows: null, scarce, slight, moderate and severe. The intensity of viral antigen staining of the ependymal cells is scored using different colours as follows: null (no colour), scarce (yellow), slight (green), moderate (blue), intense (red). (Illustration modified and reproduced with permission from Ref. [16]).

Samples of the nasal cavity and brain tissues were taken from all dead and euthanized chickens and immediately fixed in 10% buffered formalin for 24 h. Formalin-fixed nasal cavity samples were sectioned at the level of the olfactory epithelium and brain samples were cut at six different coronal levels and embedded in paraffin wax. Later, sequential 3 μm microtome sections corresponding to the following interaurals: a: 9.04 mm, b: 6.64 mm, c: 3.04 mm, d: e1.60 mm, f: -0.56 mm, and g: -3.68 mm (Figure 1) [16], were made from each paraffin block and used to perform all the immunohistochemical (IHC) studies.

### IHC to detect viral antigen in the nasal cavity and brain tissues and study its topographical distribution into the brain

An IHC for the detection of IAV nucleoprotein (NP) was performed in sections of the brain and nasal cavity from all euthanized and dead chickens in accordance with procedures previously described [17]. Briefly, paraffin-embedded samples were sectioned at 3 μm thick, dewaxed and treated with 3% H_2_O_2 _in methanol to eliminate the endogenous peroxidase. Then, sections were treated with protease at 37°C for 10 min and incubated with the primary monoclonal antibody (ATCC, HB-65, H16L-10-4R5) diluted 1:250, at 4°C overnight. After being rinsed, the samples were incubated with biotinylated goat anti-mouse IgG secondary antibody (Dako, immunoglobulins AS, Glostrup, Denmark), followed by incubation with avidin-biotin-peroxidase complex (ABC) (Thermo Fisher Scientific, Rockford, IL, USA). The reaction was developed with 3,3'-Diaminobenzidine tetrahydrochloride (DAB) (brown colour) (Sigma-Aldrich, Madrid, Spain) at room temperature (RT), followed by counterstaining with Mayer's haematoxylin. Sections from positive-control tissue blocks belonging to chicken embryos infected with the same H7N1 virus strain were included in each IHC run. Negative controls consisted of substitution of the primary antibody with phosphate buffered saline (PBS).

The distribution, intensity and pattern of H7N1 viral antigen staining in the CNS of chickens at each dpi were examined in different brain regions, including: OB, telencephalic pallium (Pall), telencephalic subpallium (SPall) (that contains the striatum (St)), hypothalamus, optic area (Och), diencephalon (that contains the prethalamus (p3), thalamus (p2), pretectum (p1), and the secondary prosencephalon (2P)), midbrain or mesencephalon, hindbrain (that contains the isthmus (Ist) and rhombencephalon (r1-6)), and the cerebellum (Cb) (Figure 1).

**Figure 2:**
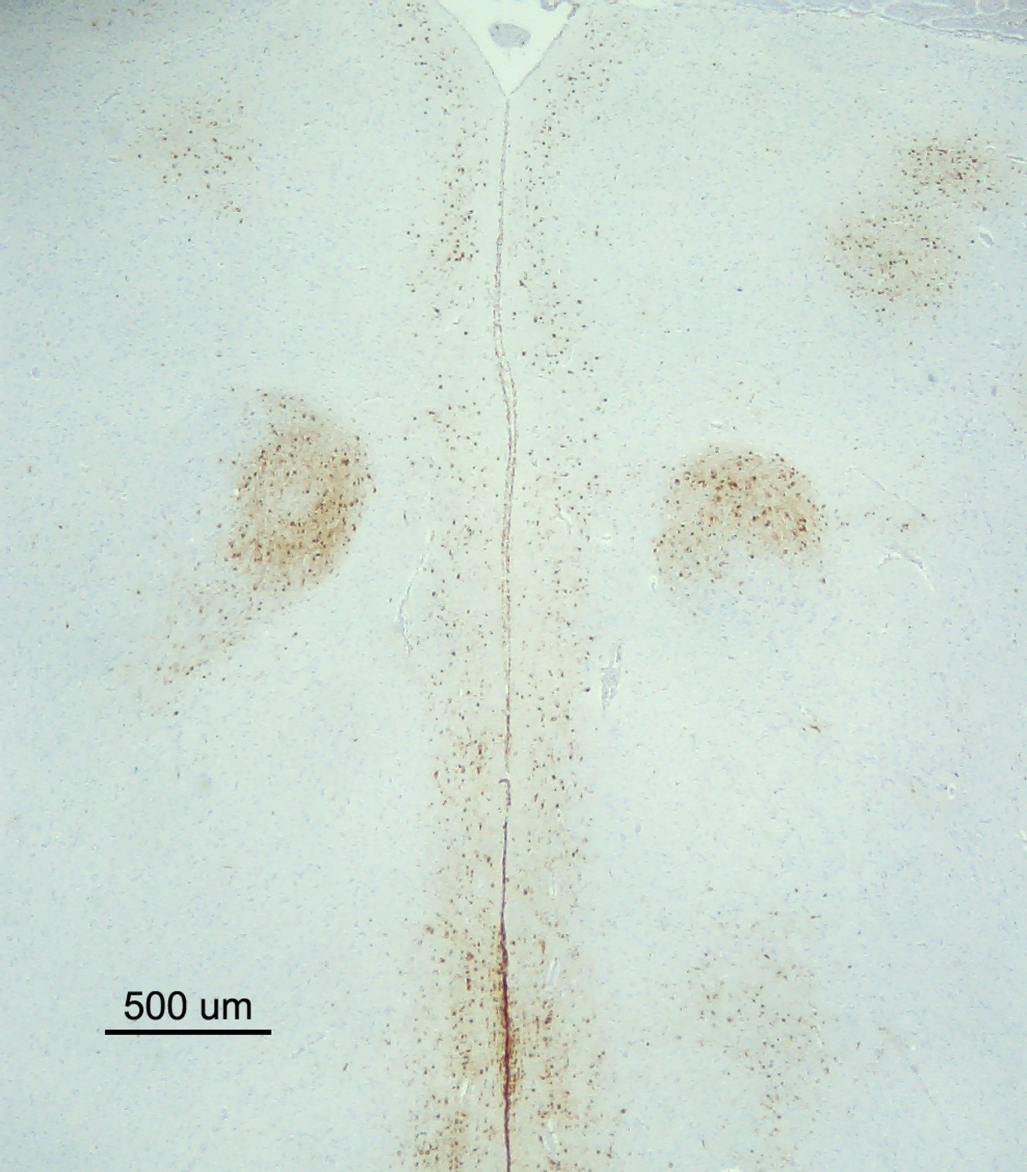
**Symmetrical and bilateral distribution of H7N1 HPAIV antigen in the brain of an infected chicken at 2 dpi**. Microphotograh showing the presence of bilateral and symmetrical viral antigen staining in the medial geniculate nucleus (MG) (nucleo ovoidalis) of the thalamus. (bar = 500 μm).

In the six coronal sections, the intensity and extent of viral antigen staining were visually determined using a scoring system, which assesses the number of positive cells including the following: neurons, glial cells (astrocytes, oligodendrocytes, microglial cells) and endothelial cells in a 10× field. To graphically represent the pattern of staining, we proceeded to quantify in each animal the number of positive cells in a variable number of 10× fields (the number of fields was variable from one to six depending on its extension) in each region described above. The following scale was used to rank the intensity of staining in each region: nil (0: no labelling detected); scarce (1: less than 20 nuclei of cells positive for viral antigen on average), slight (2: more than 20 but less than 100 positive cells on average); moderate (3: focal area of more than 100 but less than 500 positive cells on average); intense (4: focus of more than 500 positive cells on average). In a third step, we determined the arithmetic mean for each brain region and dpi, considering that we evaluated at least three birds per day. Finally, in brain regions (hypothalamus, diencephalon, mesencephalon, and hindbrain) where viral antigen was found in specific neural nuclei and symmetrically distributed (Figure 2), we evaluated the neural nuclei separately from the rest of the area, in order to better represent the viral antigen distribution. Then, the intensity of staining was represented using a heap of points labeling each specific neural nucleus (Figure 1). Finally, the topographical distribution of the viral antigen staining was graphically represented in the six coronal sections, using the Adobe Photoshop CS2 program.

The staining of ependymal cells was considered as a separate entity, and represented with different colours according to the intensity of viral antigen staining. In that way, the absence of viral antigen was not denoted, yellow was used to indicate a scarce number of positive cells (1-10 out of 100 positive cells), green for slight staining (10-30 out of 100 positive cells), blue for moderate staining (31-50 out of 100 positive cells) and red for intense staining (51-100 out of 100 cells positive).

### Lectin histochemistry for the detection of influenza virus receptors in the CNS of chickens

Lectin histochemistry was carried out on brain samples from all euthanized and dead infected and control chickens according to the protocol described by Yao et al., [18] with minor modifications. Briefly, paraffin embedded samples were sectioned at 3 μm thick, dewaxed and treated with 3% H_2_O_2 _in methanol to eliminate the endogenous peroxidase activity. Duplicated samples were rinsed with Tris-HCl buffer (TNT) and then blocked for non-specific binding with TNT plus Blocking reagent (TNB) (Perkin Elmer, Madrid, Spain) for 30 min. Samples were incubated with the biotinylated lectins *Sambucus nigrans *(SNA) (10 μg/mL) or *Maackia amurensis leukoagglutinin *(MAAII) (15 μg/mL) (Vector laboratories Inc, CA, USA) in TNB at 4°C, overnight. The MAAII lectin that preferentially binds to the Siaα2-3 Gal linkage was used to detect the avian type receptor, whereas, the SNA lectin that shows preference towards the Siaα2-6 Gal linkage was used to identify the human type receptor [19]. After washing with TNT, sections were incubated with horseradish peroxidase (HRP) 1:100 for 30 min, followed by incubation with Tyramide Signal Amplification (TSA™ Biotin System, Perkin Elmer) diluted 1:50 in buffer for 10 min and again incubated with HRP for 30 min at RT. The reaction was developed with DAB (brown colour) (Sigma-Aldrich) at RT for 30 s followed by counterstaining with Mayer's haematoxylin.

Positive controls consisted of sections of tissues where the distribution of AIV receptors has already been determined (human lungs, pig trachea, and duck lungs and intestines). Negative controls consisted of sequential tissue sections treated for 24 h with 12.5 U/μL of neuraminidase from *Clostridium perfringes *at 37°C (P0720L, New England Biolabs, MA, USA). Besides, an additional negative control consisted of substitution of the lectin by TNB buffer.

### Double IHC for the codetection of HPAIV antigen and OSN in the nasal cavity of chickens

Nasal cavity samples from 2 chickens euthanized at 2 dpi, 3 from 3 dpi and 2 from 4 dpi were double stained for the codetection of viral antigen and OSN. Briefly, paraffin-embedded tissue samples were dewaxed, cut at 3 μm thick, and blocked to eliminate the endogenous peroxidase activity using 3% H_2_O_2 _in methanol. Antigen retrieval was carried out for 30 min with proteinase K diluted 1:50 in Tris-HCl 0.05 M, pH 7.6 (S3004, Dako) at RT, and later permeabilized using triton 0.5% in phosphate buffered saline (PBS) for 10 min. Slides were blocked with 2% bovine serum albumin (BSA) (Sigma-Aldrich) diluted in 0.5% triton in PBS for 1 h at RT. The primary staining corresponded to the incubation with the antibody against IAV NP (ATCC, HB-65, H16L-10-4R5) diluted in blocking buffer and incubated overnight at 4°C. Later, tissue samples were washed three times with PBS and incubated for 1 h with alkaline phosphatase (AP) conjugate goat anti-mouse Ig (H+L) secondary antibody (1010-04, Southern Biotechnology, AL, USA), diluted in PBS. The presence of viral antigen was visualized in blue using nitro blue tetrazolium chloride 5-bromo-4-chloro-3-indoxyl (NBT-BCIP) (11 681 451 001, Roche, IN, USA).

For the second staining, samples were rinsed three times with PBS, and incubated for 1 h with the polyclonal antibody rabbit anti-human protein gene product (PGP9.5) (RA 95101, Ultraclone, Isle of Wight, UK) diluted 1:200 in PBS at RT. After that, tissue sections were incubated for 1 h with the biotinylated secondary antibody, followed by incubation with ABC and visualization of the reaction using DAB (Thermo Fisher Scientific, Rockford, IL, USA), obtaining a brown staining where OSN were detected. Samples were not counterstained. Negative controls consisted of incubation of a sequential sample with PBS instead of the primary antibodies. Unspecific binding of both secondary antibodies was discarded, incubating them with the contrary primary antibody.

### Combined lectin and IAV IHC staining for the codetection of IAV antigen and Siaα2-6 Gal receptors in the CNS of chickens

Brain sections of two infected and euthanized chickens obtained for each dpi and an equal number of sections of sham-inoculated control tissues were double stained for the detection of Siaα2-6 Gal receptors and IAV nucleoprotein. To that end, SNA lectin staining was performed as previously described and visualized in brown colour using DAB. Later, samples were rinsed three times with tris-buffer saline solution (TBS), treated with proteinase K (Dako) and blocked with 2% BSA diluted in TBS (Sigma-Aldrich). Samples were incubated with the IAV antibody (ATCC, HB-65, H16L-10-4R5) diluted 1:1000, overnight at 4°C and visualized by incubation with goat anti-mouse Ig (H+L)-PA (1010-04, AL, Southern Biotechnology) for 1 h at RT and NBT-BCIP for 10 min (11 681 451 001, Roche). The positive reaction was visualized in blue colour. Samples were not counterstained. Negative controls included substitution of the IAV antibody or the lectin per PBS and incubation of the lectin with the biotinylated anti-mouse antibody.

### RT-qPCR for the detection of viral RNA in CSF and blood

The RT-qPCR technique used to quantify the viral RNA copies have been thoroughly described previously [20]. Briefly, viral RNA was extracted from each sample using QIAamp viral mini kit (Qiagen, Hilden, Germany). The RNA was eluted in 40 μL and amplified by one-step RT-qPCR for the detection of a highly conserved region of the matrix (*M*) gene of IAV using the previously described primers [21]. This procedure uses an internal positive control (IPC) to avoid false negative results due to RT-qPCR inhibitors.

## Results

### Clinical signs, mortality and gross lesions

No clinical signs and gross lesions were observed at one dpi. Eight out of 14 infected chickens showed depression, prostration, ruffled feathers and respiratory distress at 2 dpi. Conjunctivitis was noted in one out of 14 chickens on this day. At necropsy, only pulmonary congestion was observed in 2 out of 3 chickens at 2 dpi. At 3 dpi, 4 chickens were found dead, whilst, severe depression, inactivity and neurological signs were additionally observed in 3 out of 11 chickens. Macroscopic lesions of petechial haemorrhages in the unfeathered skin of the leg and comb, as well as in skeletal muscles and pancreas were also recorded in 2 out of the 3 euthanized chickens and in all chickens found dead. At 4 dpi one chicken was found dead, whereas, similar clinical presentation with prostration, dyspnoea and neurological signs of profound depression, tremors and loss of balance were observed in the rest of the chickens. Gross lesions also consisted of petechial to ecchymotic haemorrhages in the comb and legs, pallor of the kidney with accentuated lobular surface, atrophy of the bursa of Fabricius, petechial haemorrhages in the serosa on the proventricular serosa, and multiple petechial to ecchymotic haemorrhages in the pancreas. There were no clinical signs, mortality or gross lesions in the control non-infected chickens.

### Histological lesions and pattern of H7N1 HPAIV staining and topographical distribution in the CNS of infected chickens

No viral antigen was detectable in any region of the brain of infected chickens at 1 dpi. Microscopic lesions of perivascular oedema, endothelial cell hypertrophy, focal and focus of malacia associated to moderate gliosis were mainly seen in the gray matter of the brain at 2 dpi. Additionally, at 3 and 4 dpi, focal haemorrhages and coalescent foci of malacia were observed. Viral antigen was detected from 2 to 4 dpi in all 6 chicken brain coronal sections, albeit with different intensities. Euthanized and dead chickens showed similar patterns and intensities of staining. Viral antigen staining was detected as dark brown chromogen deposition predominantly in the nuclei of neurons, glial cells, endothelial and ependymal cells, and less commonly, as granular cytoplasmatic staining in neurons, endothelial and ependymal cells. In addition, granular or linear brown staining in the neuropil was observed and interpreted as positive neuronal axons and dendrites, and cytoplasmatic prolongations of glial cells. Overall, positive viral antigen staining was found mainly in the gray matter but there were small sporadic positive foci in the white matter. Altogether, viral antigen positive cells and neuropil staining was found in isolated or multiple foci that in severe cases coalesced in large round to irregular foci. These foci were randomly distributed in the telencephalic pallium (medial, dorsal, lateral, and ventral pallium - MPall, DPall, LPall and VPall, respectively), SPall, and St, and their location varied among chickens. In contrast, symmetrical and bilateral viral antigen distribution was observed in the Mesencephalon, hypothalamus, diencephalon, and r1-6, where the staining was restricted to particular neural nuclei (Figures 1 and 2). Staining in the choroid plexus was scarce and mainly noticed in the nuclei of epithelial choroid plexus cells, although cytoplasmatic staining was also observed.

The topographical distribution and intensity of viral antigen staining, thoroughly examined, quantified and represented in Figure 1, shows how the virus spreads into the CNS of chickens. According to the viral antigen neuroanatomical distribution, the most rostral sections of the brain displayed less antigen staining in comparison with the most caudal sections, with the exception of the rhombencephalon where the staining was weak. The intensity of the staining increased with time, reaching a maximum at 4 dpi. In particular, viral antigen staining was scarcely detected in cells of the OB at 2 and 3 dpi, whereas, a slight increase was observed at 4 dpi. Likewise, weak viral antigen staining was observed in the telencephalic pallium (DPall, LPall, and VPall) and SPall of the most rostral regions at 2 dpi. At 3 dpi, moderate staining was observed in the LPall and VPall, while at 4 dpi the viral antigen staining was intense in the pallium (DPall, LPall and VPall) and SPall. Furthermore, the most rostral periventricular regions and ependymal cells showed scarce to minor staining (labelled in yellow) at 2 and 3 dpi, whereas, at 4 dpi, moderate staining was observed in these most rostral periventricular regions and on ependymal cells (labelled in blue).

Viral antigen staining in the intermediate section of the telencephalic pallium, essentially in the MPall, DPall, LPall, and St, was very weak at 2 dpi, and moderate at 3 dpi. However, the VPall showed little increase in viral antigen staining at 2 dpi and intense staining at 3 dpi. The most caudal section of the telencephalic pallium showed high viral antigen staining, being moderate at 2 dpi and intense at 3 dpi. At 4 dpi, the amount of viral antigen staining on the intermediate and caudal sections of the telencephalic pallium was intense, with formation of large coalescent positive viral antigen areas. Slight to moderate viral antigen staining was observed in the hypothalamus, thalamus (p2), prethalamus (p3), and secondary prosencephalon (2P) at 2 and 3 dpi, respectively. Furthermore, it was almost restricted to the following neural nuclei: dorsolateral nucleus of the thalamus (DLA), pregeniculate nucleus (PG), medial geniculate nucleus (MG), the rotundus nucleus of the thalamus (Rot), anterior pretectal nucleus (APT) and tectal gray-superficial stratum (TGS). On these nuclei, the viral antigen was also found bilaterally and symmetrically. Similar intensity of staining was observed on ependymal cells, being slight at 2 dpi (labelled in green) and moderate at 3 dpi (labelled in blue). At 4 dpi, bilateral and symmetrical viral antigen staining was found in the same neural nuclei observed at 2 and 3 dpi, with an evident increase in the intensity of the staining. Additionally, at 4 dpi viral antigen staining was also detected in the following neural nuclei: lateral hypothalamic area (LH), dorsomedial anterior nucleus of the thalamus (DMA), subthalamic nucleus (STh) in the mammilary region, ventromedial hypothalamic nucleus (VMH) and the r3 lateral vestibular nucleus, ventral part (r3LVeV). Viral antigen positive staining was scored as moderate for the periventricular regions and ependymal cells at 4 dpi (labelled in blue).

In the mesencephalon, slight staining was observed at 2 and 3 dpi, whereas moderate staining was found at 4 dpi. On these regions, the viral antigen staining was also found bilateral and symmetrically and almost restricted to the following nuclei: the torus semicircularis (ToS), red nucleus, magnocellular part (RMC), and the mesencephalic substancia nigra, compact part (mSNC). The intensity of staining in the mesencephalic periventricular zone and ependymal cells increased in severity from slight (labelled in green) to intense (red) from 2 to 4 dpi, respectively.

In the cerebellum, high virus affinity was noted for the Purkinje cells and their cytoplasmic processes in the molecular layer. Less intensity of staining was observed in the granular layer. The viral antigen staining in the cerebellum was scored as intense at 2, 3 and 4 dpi, but their distribution varied, being found as single individual foci at 2 dpi and large coalescent viral antigen positive areas at 3 and 4 dpi. Moderate to intense bilateral staining was observed in the rhombencephalon from 2 to 4 dpi, respectively. Bilateral and symmetrical distribution was observed in the following rhombencephalic nuclei: medial cerebellar nucleus (Med), r2 superior vestibular nucleus (r2SuVe), the oral part of the spinal trigeminal nucleus (Sp50). In the rhombencephalon, the viral antigen staining on ependymal cells was weak at 2 dpi (labelled in green) and increased on severity at 3 and 4 dpi, where moderate staining was found (blue).

Epithelial cells of the choroid plexus in the lateral, third and fourth ventricles showed less intensity of viral antigen staining in comparison with the ependymal cells, being scarce at 2 dpi and slight at 3 and 4 dpi. In the same way, although cells of the leptomeninges were negative, subpial astrocytes in the glial limitants in the intermediate and caudal sections of the brain showed positivity in each dpi, but particularly at 4 dpi.

In general, the most commonly affected neural nuclei were the following: the PG (found positive in 12 out of 14 chickens) and Rot in the thalamus (11 out of 14 chickens), ToS in the mesencephalon (9 out of 14 chickens), and APT in the pretectum (8 out of 14 chickens).

It was not possible to evaluate all circumventricular organs (CVOs) in each chicken every dpi. However, at 2 dpi, slight viral antigen staining was observed in the striopallidal organ (SPO) (4 out of 4 chickens), area postrema (AP) (in one chicken), vascular organ of the lamina terminalis (VOLT) (in one chicken) (Additional file 1) and the lateral septal organ (LSO) (one chicken). The subcommisural organ (SCO) was found negative in one chicken. Whereas at 3 dpi, the SPO (4 out of 5 chickens), SCO (3 out of 3 chickens), area postrema (AP) (one chicken), median eminence (ME) (2 chickens) and the VOLT (one chicken) and the LSO (one chicken) showed moderate staining. Moderate to intense viral antigen staining was observed in SCO, ME and SPO at 4 dpi (one chicken).

### Distribution of influenza virus receptors in the CNS of control and infected chickens

**Figure 3:**
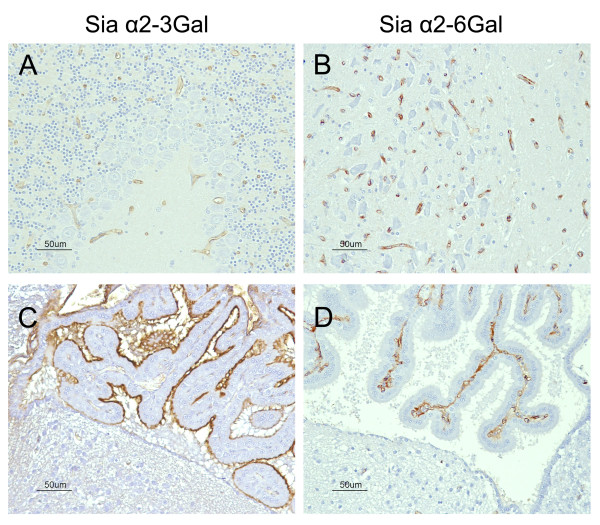
**Detection of Siaα2-3 Gal and Siaα2-6 Gal IAV receptors in the brain of chickens using the lectins MAAII and SNA, respectively**. Slight staining for Siaα2-3 Gal receptors was found on endothelial cells (A) and ependymal cells, whereas the apical membrane of choroid plexus cells showed an abundant presence of Siaα2-3 Gal receptors (C). Intense staining for Siaα2-6 Gal receptors was observed on endothelial cells in the brain and choroid plexus cells. (bar = 50 μm).

**Figure 4:**
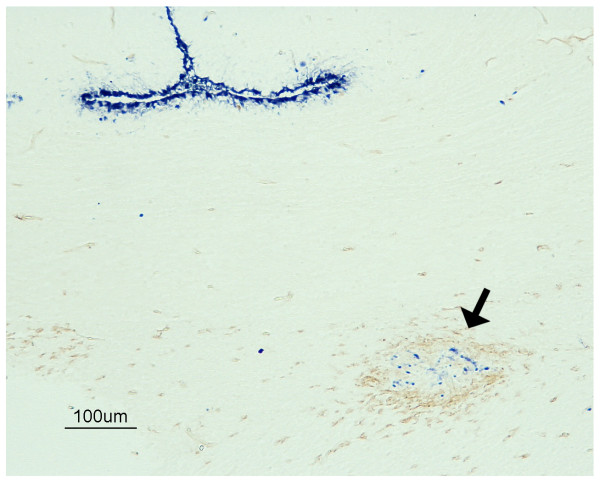
**Codetection of viral antigen and Siaα2-6 Gal receptors in the brain of an H7N1 HPAIV infected chicken at 3 dpi**. Double immunohistochemistry showing the focus of SNA granular staining (arrow) in correlation with the presence of influenza virus antigen detected in the optic chiasm of infected chicken. Influenza virus antigen was abundant also on ependymal cells (bar = 100 μm).

Siaα2-3 Gal receptor was detected in the apical surface of choroid plexus cells and ependymal cells of the lateral and fourth ventricle, whereas only slight staining was detected on endothelial cells (Figure 3a, c). There were no differences in the pattern and intensity of staining with the MAAII lectin between healthy and infected chickens, also among different dpi. Neither were there any differences in staining pattern observed with both lectins between dead and euthanized chickens. Siaα2-6 Gal receptors were observed in the luminal border of endothelial cells distributed throughout the brain parenchyma, choroid plexus, and meninges (Figure 3b, d). Differences in the pattern of staining with this lectin (SNA) were detected between healthy and HPAIV infected chickens. In infected chickens, foci of granular SNA positive staining in the neuropil were observed adjacent to SNA-positive endothelial cells. These foci were found randomly distributed in all regions of the brain of infected chickens at 3 and 4 dpi, being more frequent in the most affected chickens (Figure 4).

### Co-detection of Siaα2-6 Gal receptors and HPAIV in the CNS of infected chickens

In order to understand the nature of the granular focus of SNA staining observed in the brains of chickens infected with this HPAIV, a double IHC was performed in sections of HPAIV infected and control chickens. Interestingly, the foci of granular SNA lectin deposition were frequently observed associated with the presence of viral antigen staining and malacia at 3 and 4 dpi (Figure 4). However, granular SNA lectin deposition was not found in all viral antigen positive areas.

### Codetection of avian influenza virus antigen and olfactory sensory neurons (OSN) in the nasal cavity of infected chickens

**Figure 5:**
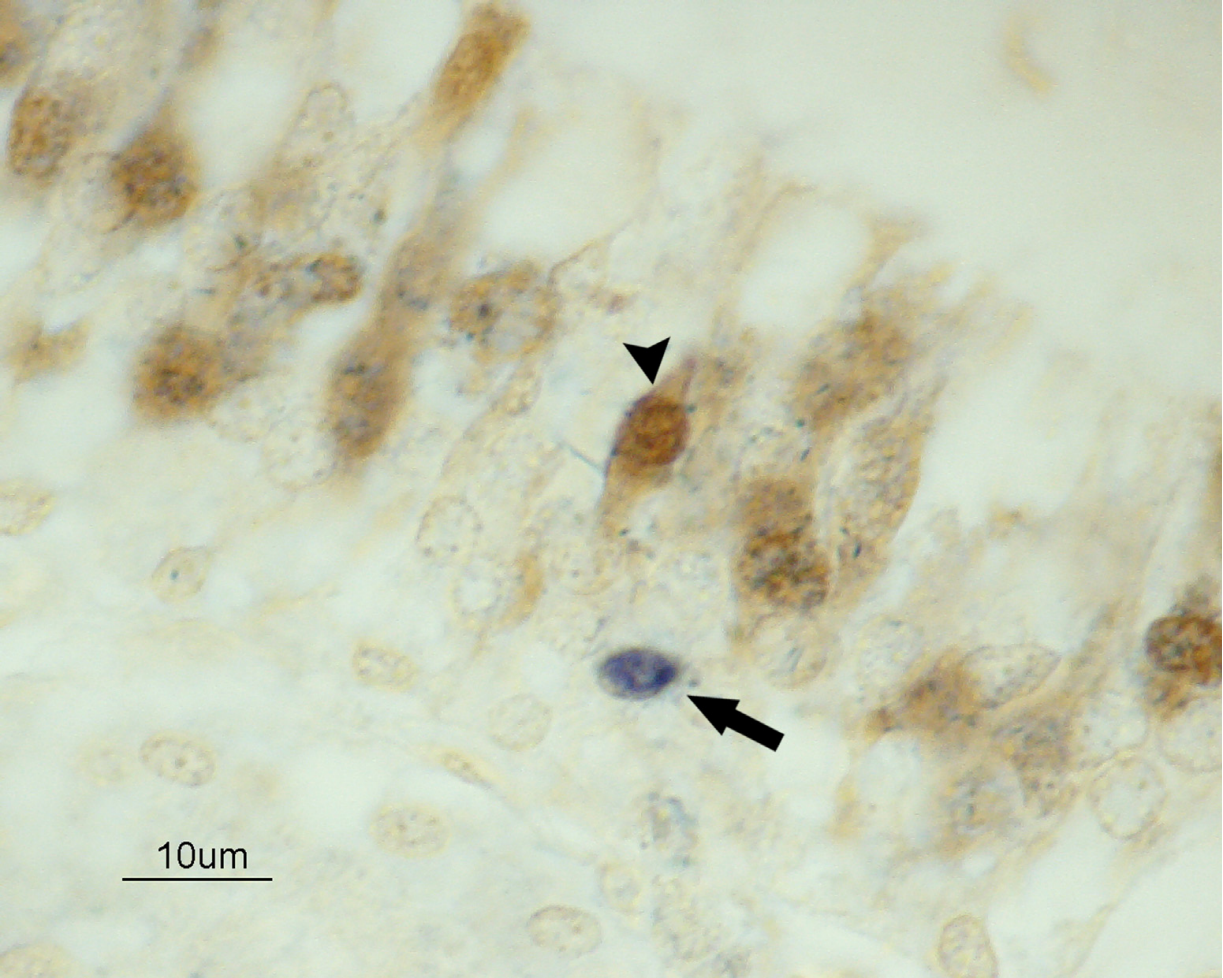
**Codetection of IAV antigen in OSN of the nasal cavity in a chicken infected with H7N1 HPAIV, at 3 dpi**. Double immunohistochemistry showing OSN labelled with PGP9.5 (arrowhead). Viral antigen was found in supporting (arrow) and basal cells of the nonsensory epithelium (not stained), but not in OSN. (bar = 10 μm).

PGP9.5 antigen was detected in the nucleus and cytoplasm of olfactory neurons in all chickens. Viral antigen staining was sporadically observed in the nuclei of cells of the olfactory epithelium of chickens infected with H7N1 HPAIV from 2 to 4 dpi. Viral antigen staining did not colocalize with the PGP9.5 antigen in any chicken (Figure 5).

### Detection of viral RNA in blood and CSF

Viral RNA was detectable in blood and CSF as early as 1 dpi. At this day, 5.03 log_10 _viral RNA copies per mL in the blood and 1.36 log_10 _viral RNA copies per μL in the CSF were reported. At 3 dpi, the viral load increased to 9.21 log_10 _viral RNA copies per mL in blood and 5.39 log_10 _viral RNA copies per μL in CSF.

## Discussion

Although HPAIV neuroinvasiveness and neurovirulence are considered to be one of the main factors leading to the fatal course of infection in birds [22], few detailed studies on viral neuropathogenesis have been conducted in chickens. Previous experimental studies carried out on different species have suggested that IAV could enter into the CNS through one or more of these three pathways: haematogenous [5,23], olfactory [24,25] and neural routes [7,26].

Earlier we reported sparse detection of H7N1 HPAIV in olfactory epithelium at 7 dpi [27]. Therefore, we were interested in elucidating whether OSN could provide free access for the virus to enter the CNS. Several neurotropic viruses, such as herpes simplex virus, Borna disease virus, rabies virus, vesicular stomatitis virus, parainfluenza virus, mouse hepatitis virus, and Venezuelan equine encephalitis virus have been proven to use the olfactory pathway to enter into the CNS [24,28-30]. However, in the present study, OSN were negative for viral antigen during the first four days of evaluation, confirming that these cells are not a major target for the virus. Viral antigen positive cells in the olfactory epithelium corresponded to basal or supporting cells. In addition, according to the topographical distribution of the viral antigen, the relevance of the olfactory pathway for virus invasion into the CNS could be considered as negligible or absent, because scarce to slight staining was found in the OB and the most cranial regions of the brain at 2 dpi (Additional file 1).

IAV antigen has also been detected in peripheral nerves, plexus and ganglia of thoracic and enteric tissues in turkeys, Japanese quails, pheasants, partridges, domestic ducks and house sparrows infected with HPAIV [31-35]. In a previous work, we reported the presence of H7N1 HPAIV antigen in branches of the trigeminal nerve of chickens at 7 dpi [27]. However, based on the present results, the neural pathway does not seem to play an important role in IAV neuropathogenesis in birds, because neural nuclei associated with cranial nerves, such as the vestibular nerve (r2SuVe, r3LVeD, r4LVeD) or the trigeminal nerve (Sp50) were only sporadically positive at early stages of infection, nor was there caudal to cranial increase in viral antigen staining observed in any of the chicken brains examined.

Early endothelial infection and viraemia are key events during HPAIV infection in birds [5,36]. The viraemia determines the spreading of this H7N1 HPAIV to different organs and final entrance to the CNS [4,37], similarly to other viral infections, such as canine distemper virus [38] and human immunodeficiency virus [28]. Several results obtained in the present study support the hypothesis that the bloodstream is the main route of entry and early dissemination of HPAIV into the brain. Firstly, the detection of viral RNA in blood at one dpi confirms the capacity of this virus to induce viraemia very early during the course of the infection. Secondly, the detection of Siaα2-3 Gal and Siaα2-6 Gal receptors on CNS endothelial cells and, moreover, the finding of viral antigen in brain capillary endothelial cells and the surrounding nervous parenchyma as early as 2 dpi, corroborates the permissiveness of this cell type for HPAIV entrance and replication and further support the haematogenous neuroinvasion hypothesis. In addition, the widespread distribution of the viral antigen during the early stages of infection follows a pattern consistent with a haematogenous viral spreading [39].

Neuroinvasion through the haematogenous pathway implies that the virus could cross the blood brain barrier (BBB) localized in the brain endothelial cell and the blood-CSF barrier, confined to the choroid plexus [40]. To overcome these barriers and disseminate into the CNS, viruses and other infectious agents must disrupt the BBB in the brain parenchyma and meninges or cross the fenestrated endothelium located in the choroid plexus and CVOs [41]. Detection of abundant and widespread viral antigen in brain capillary endothelial cells and astrocytes forming the glia limitants suggest this H7N1 HPAIV could be able to disrupt the BBB and infect the brain parenchyma. However, the detection of viral RNA in CSF of chickens at one dpi, indicates that disruption of the blood-CSF barrier occurs subsequently to the establishment of viraemia, as has also been demonstrated for other neurotropic viruses (HIV, simian and feline immunodeficiency viruses) [42-45]. Epithelial and endothelial cells of the choroid plexus as well as ependymal cells expressed Siaα2-3 Gal receptors, therefore it is not surprising to detect viral antigen as early as 2 dpi, on these cells. It has to be pointed out that the viral antigen was very abundant in ependymal cells, which could be a consequence of further replication of H7N1 HPAIV, amplification and dissemination of the virus by means of the CSF along the ventricular system and in the subpial parenchymal areas.

The CVOs can be portals of entry of infectious pathogens, such as *trypanosoma brucei*., bovine spongiform encephalopathy and Scrapie, into the CNS [46,47]. Similarly, in this study we observed viral antigen in CVOs (SPO, AP, VOLT, LSO) early during the infection, which is in agreement with the expected facility of a virus that causes viraemia to enter into brain areas lacking a real BBB. However, we did not observe dissemination of the viral antigen from CVOs to the surrounding brain regions. Instead, the staining was almost restricted to these brain areas, indicating that these organs might contribute to virus entry and spreading in the CNS, but is not the main route of entry.

Interestingly, bilateral and symmetrical areas of positive viral detection were observed in the diencephalon, mesencephalon, and rhombencephalon, especially in the Rot (p2), PG (p3), ToS (mesencephalon), and APT nuclei (p1). Detection of bilateral and symmetrical lesions in the brain has also been described in humans suffering from influenza-associated encephalopathies (IAE) [48,49]. In these conditions, it has been suggested that regional differences in blood flow and myelinisation could determine the distribution of lesions [50]. Based on our results, the symmetrical and bilateral distribution of the viral antigen in the CNS of chickens could be explained by the distribution of the blood vessels in the brain and the direct infection of selected periventricular nuclei.

Few studies have been conducted to investigate the presence of influenza virus receptors in the brain of chickens and other species. In spite of that, both receptors have been reported on endothelial cells of humans, pigs, rats and chicken embryos; however, they are not specific to the brain [11,18,51,52]. In chickens, previous investigations have failed to detect avian influenza receptors in brain endothelial cells [53], which could be explained by differences in the sensitivity between the histochemical techniques and specially due to the signal amplification system used for the IHC in the present study.

Similarly, there are no studies comparing the presence of both receptors in infected and healthy chickens, which as demonstrated in this study was altered. Increased detection of SNA staining in association with the presence of necrotic foci and viral antigen was observed in infected chickens. Local up-regulation of Siaα2-6 Gal glycotopes has been reported under different experimental inflammatory conditions [54-56]. For example, increase Siaα2-6 and Siaα2-3 glycotopes have been detected in respiratory cells of ferrets infected with H1N1 influenza virus [55], mice affected with asthma [54] and in serum of mice injected with turpentine oil to induce inflammation [56]. Similarly, an increased expression of mRNA of α2-6 and α2-3 sialyltransferases have been observed in rat liver after inflammation [57], and human tracheal cells and bronchial mucosa stimulated with tumour necrosis factor-α (TNFα) [58]. However, considering that most acute-phase proteins and other inflammation-sensitive glycoproteins contain sialic acids [56,59,60], the increased expression of Siaα2-6 Gal observed in the brain of infected chickens needs careful interpretation and further investigation to understand its role in IAV neuropathogenicity.

In summary, the results obtained in this study indicate that the most likely pathway for entry of the H7N1 HPAIV into the chicken brain is the haematogenous route. This is supported by the fact that brain endothelial cells, choroid plexus and ependymal cells hold large numbers of IAV receptors in their surface, which could play an important role, together with the CSF, in the entry and early dissemination of the virus. Further studies are needed to explain the exact mechanism leading to the disruption of the BBB by IAV.

## List of Abbreviations

(AIV): Avian influenza virus; (AP): Alkaline phosphatase; (APT): anterior pretectal nucleus; (AP): area postrema; (ABC): avidin-biotin-peroxidase complex; (BBB): blood brain barrier; (BSA): bovine serum albumin; (CNS):central nervous system; (Cb): Cerebelum; (CSF): cerebrospinal fluid; (CVOs): circumventricular organs; (DAB): 3,3'-Diaminobenzidine tetrahydrochloride; (DLA): dorsolateral anterior nucleus; (DMA): dorsomedial anterior nucleus of the thalamus; (DPall): dorsal pallium; (HPAIV): highly pathogenic avian influenza virus; (HRP): horseradish peroxidase; (IAE): influenza associated encephalopathies; (IHC): immunohistochemistry; (LH): lateral hypothalamic area; (LPall): lateral pallium; (LSO): lateral septal organ; (MAAII): *Maackia amurensis leukoagglutinin *; medial cerebellar nucleus; (Med):(LPAIV): low pathogenic avian influenza virus; (ME): median eminence; (MG): medial geniculate nucleus; (MPall): medial pallium; (mSNC): mesencephalic substancia nigra, compact part; (NBT-BCIP): nitro blue tetrazolium chloride 5-bromo-4-chloro-3-indoxyl; (NP): nucleoprotein; (OSN): olfactory sensory nervous; (Pall): telencephalic pallium; (PBS): phosphate buffered saline; (PG): pregeniculate nucleus; (PGP9.5): protein gene product; (p2): thalamus; (p3): prethalamus; (2P): secondary prosencephalon; (RMC): red nucleus magnocellular part; (RT-qPCR): quantitative real-time reverse transcription-polymerase chain reaction; (Rot): rotundus nucleus of the thalamus; (RT): room temperature; (r2SuVe): r2 superior vestibular nucleus; (r3LVeV): r3 lateral vestibular nucleus, ventral part; (SNA): *Sambucus nigrans *; optic area; (Och):(Sp50): spinal trigeminal nucleus, oral part; (SCO): subcommisural organ; (SPall): telencephalic subpallium; (SPO): striopallidal organ; (STh): subthalamic nucleus; (TBS): Tris-buffer saline solution; (TGS): tectal gray-superficial stratum; (TNT): Tris-HCl buffer; (TNB): TNT plus blocking buffer; (ToS): torus semicircularis; (VOLT): vascular organ of the lamina terminalis; (VPall): ventral pallium; (VMH): ventromedial hypothalamic nucleus.

## Competing interests

The authors declare that they have no competing interests.

## Authors' contributions

NB prepared the viruses used in this study. AC, RV, AR and NM participated in the daily monitoring of the clinical signs and the sampling of the animals during the whole experimental period. AC, JVA, RV, RD, AR and NM performed the necropsies and the tissue sampling. AC and RV performed the histopathology and immunohistochemistry techniques of the necropsy tissues. AC and NM carried out the histopathological examinations. NB and RR carried out the avian influenza virus quantitation by RT-qPCR. AC, NB, JVA, AR, AD and NM conceived the study and participated in its design and coordination. All authors read and approved the final manuscript.

## Supplementary Material

Additional file 1 **Schema representing the mechanism by which viruses could enter into the CNS and photographs showing the possible routes used by the H7N1 HPAIV in chickens**. Haematogenous route includes: 1. Disruption of the BBB at the level of parenchyma endothelial cells and direct entry of the virus into the brain. Microphotography 1. shows viral antigen staining on vascular endothelial cells in the molecular and granular layer of the cerebellum. (bar = 25 μm). 2. Disruption of the blood-CSF-barrier with infection of the CSF. Microphotography 2. shows viral antigen staining in vascular endothelial cells and choroid plexus epithelial cells. 3. Disruption of the BBB at the level of the CVOs. (bar = 25 μm). Microphotography 3. shows viral antigen staining of ependymal cells and glial cells in the vascular organ of the lamina terminalis (VOLT). (bar = 50 μm) 4. Disruption of vascular endothelial cells in the meninges with infection of the CSF. Microphotography 4. shows viral antigen staining of vascular endothelial cells and astrocytes in the glia limitants. (bar = 25 μm). 5. Nervous route: includes the olfactory pathway and other cranial nerve pathways. (5a) Illustration showing the anatomical position of the OB in chickens. (5b). Schematic diagram of the olfactory epithelium and OB, showing the distribution of the OSN. Microphotography 5c. shows viral antigen staining of a few glial cells and neurons in the olfactory bulb of a chicken infected with H7N1 HPAIV at 3 dpi. (bar = 50 μm) (Illustration modified and reproduced with permission from Ref. [61]). Click here for file
