# Supplementary material for: Neuropathogenesis of a highly pathogenic avian influenza virus (H7N1) in experimentally infected chickens
Source: Vet Res. 2011 Oct 7;42(1):106. doi: 10.1186/1297-9716-42-106 (PMC3199250; doi:10.1186/1297-9716-42-106)
Supplement: Additional file 1 — Schema representing the mechanism by which viruses could enter into the CNS and photographs showing the possible routes used by the H7N1 HPAIV in chickens. Haematogenous route includes: 1. Disruption of the BBB at the level of parenchyma endothelial cells and direct entry of the virus into the brain. Microphotography 1. shows viral antigen staining on vascular endothelial cells in the molecular and granular layer of the cerebellum. (bar = 25 μm). 2. Disruption of the blood-CSF-barrier with infection of the CSF. Microphotography 2. shows viral antigen staining in vascular endothelial cells and choroid plexus epithelial cells. 3. Disruption of the BBB at the level of the CVOs. (bar = 25 μm). Microphotography 3. shows viral antigen staining of ependymal cells and glial cells in the vascular organ of the lamina terminalis (VOLT). (bar = 50 μm) 4. Disruption of vascular endothelial cells in the meninges with infection of the CSF. Microphotography 4. shows viral antigen staining of vascular endothelial cells and astrocytes in the glia limitants. (bar = 25 μm). 5. Nervous route: includes the olfactory pathway and other cranial nerve pathways. (5a) Illustration showing the anatomical position of the OB in chickens. (5b). Schematic diagram of the olfactory epithelium and OB, showing the distribution of the OSN. Microphotography 5c. shows viral antigen staining of a few glial cells and neurons in the olfactory bulb of a chicken infected with H7N1 HPAIV at 3 dpi. (bar = 50 μm) (Illustration modified and reproduced with permission from Ref. [61]). [file 1297-9716-42-106-S1.PDF]

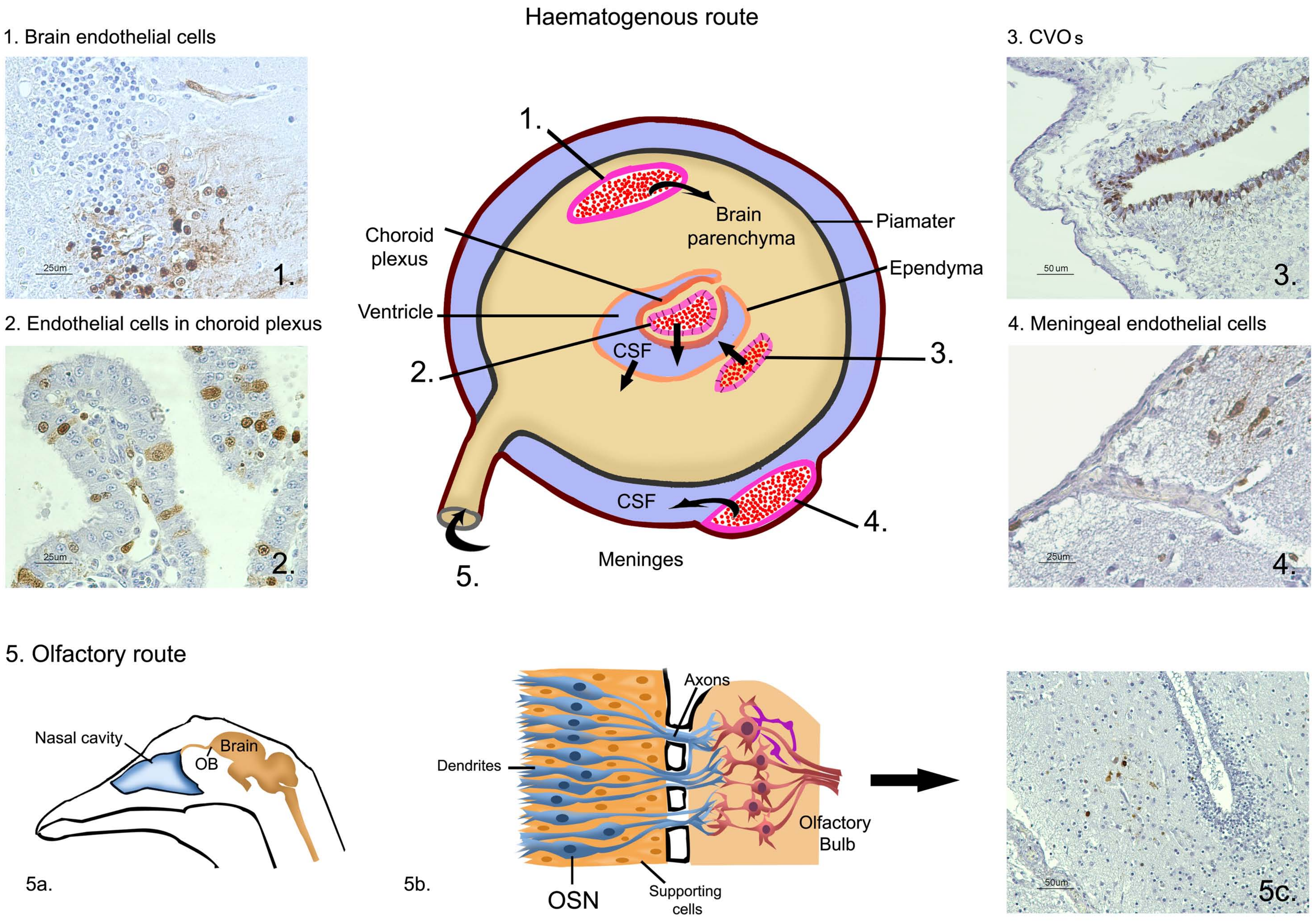

Haematogenous route
1. Brain endothelial cells
25um
1.
2. Endothelial cells in choroid plexus
25um
2.
3. CVO s
50 um
3.
4. Meningeal endothelial cells
25um
4.
1.
Brain
parenchyma
Piamater
Choroid
plexus
Ependyma
Ventricle
CSF
2.
3.
CSF
4.
5.
Meninges
5. Olfactory route
Nasal cavity
Brain
OB
5a.
Axons
Dendrites
Olfactory
Bulb
OSN
Supporting
cells
5b.
50um
5c.
